# Use of artificial neural networks to identify the predictive factors of extracorporeal shock wave therapy treating patients with chronic plantar fasciitis

**DOI:** 10.1038/s41598-019-39026-3

**Published:** 2019-03-12

**Authors:** Mengchen Yin, Junming Ma, Jinhai Xu, Lin Li, Guanghui Chen, Zhengwang Sun, Yujie Liu, Shaohui He, Jie Ye, Wen Mo

**Affiliations:** 1 Department of Orthopaedics, LongHua Hospital, Shanghai University of Traditional Chinese Medicine, Shanghai, China; 2 Department of Bone Tumor Surgery, Changzheng Hospital, Second Military Medical University, Shanghai, China; 3 Department of Orthopedics, Peking University Third Hospital, Beijing, China

## Abstract

The purpose of our study is to identify the predictive factors for a minimum clinically successful therapy after extracorporeal shock wave therapy for chronic plantar fasciitis. The demographic and clinical characteristics were evaluated. The artificial neural networks model was used to choose the significant variables and model the effect of achieving the minimum clinically successful therapy at 6-months’ follow-up. The multilayer perceptron model was selected. Higher VAS (Visual Analogue Score) when taking first steps in the morning, presence of plantar fascia spur, shorter duration of symptom had statistical significance in increasing the odd. The artificial neural networks model shows that the sensitivity of predictive factors was 84.3%, 87.9% and 61.4% for VAS, spurs and duration of symptom, respectively. The specificity 35.7%, 37.4% and 22.3% for VAS, spurs and duration of symptom, respectively. The positive predictive value was 69%, 72% and 57% for VAS, spurs and duration of symptom, respectively. The negative predictive value was 82%, 84% and 59%, for VAS, spurs and duration of symptom respectively. The area under the curve was 0.738, 0.882 and 0.520 for VAS, spurs and duration of symptom, respectively. The predictive model showed a good fitting of with an overall accuracy of 92.5%. Higher VAS symptomatized by short-duration, severer pain or plantar fascia spur are important prognostic factors for the efficacy of extracorporeal shock wave therapy. The artificial neural networks predictive model is reasonable and accurate model can help the decision-making for the application of extracorporeal shock wave therapy.

## Introduction

Plantar fasciitis is the most common complaint of patients visiting foot and ankle specialists^1,2^. Plantar fasciitis often causes pain in the heel or in the foot arch. The pain becomes most evident when the patient stands up ready to walk or has walked, run, or stood for a long period. Usually sharp at first, the pain may dwindle or relieve when the patient moves in a light manner. One or both feet can be affected^3,4^. The etiology of plantar fasciitis is multifactorial and poorly understood^5^. Plantar fasciitis is more likely to inflict people who frequently run or perform high-impact activities like jumping, dancing or athletic running^6^. Plantar fasciitis is supposed to be caused by biomechanical overstress of calcaneal tuberosity^7^. Its biomechanical etiology usually involves the windlass mechanism and tension of the plantar fascia in both stance and gait^8^. People who are overweight and have tight calf muscles, high-arched feet, or flat feet are at risk.

The diagnosis of plantar fasciitis is based on patient’s medical history and physical examination performed by a health care practitioner^9^. Patients usually present with plantar heel pain when bearing weight, particularly on getting up in the morning or after a period of rest. Then the pain severity decreases during a few minutes and increases as the feet continue to bear the body weight^10,11^. Imaging examination or other routine tests are not needed to make the diagnosis, but they are sometimes useful to rule out other causes of foot pain, such as broken bone fracture or nerve injury.

The symptoms can be alleviated by conservative treatments, including stretching the plantar fascia and the calf muscles, wearing shoes with proportional arch, taping the bottom of the feet or using shoe inserts stretching and corticosteroid injection. If a patient’s heel pain persists for more than one month, chronic recalcitrant plantar fasciitis is suspected^12^. Extracorporeal shockwave therapy (ESWT) is an alternative to surgery and ineffective conservative treatment for recalcitrant heel pain syndrom, approved in 2000 by the Food and Drug Administration^13–15^. It is widely used because it achieves a fast recovery without the necessity of reducing weight bearing or immobilization. ESWT stimulates the wound healing cascade, turns chronic damage into acute damage and initiates the normal wound healing process by pressing high intensity waves into the body^13,16^.

Previous studies have reported ESWT realizes a success rate of 34% to 88% for plantar fasciitis^17–19^. Currently, controversy has emerged regarding the relationship between a patient’s characteristics and ESWT’s effectiveness. ESWT’s effectiveness depends on several factors, which include disease symptoms and an array of therapeutic parameters. As far as we know, no clinical study has been investigating prognostic factors for ESWT therapeutic outcomes. Therefore, it is important to determine how a patient’s individual demographic characteristics, physical signs, pain duration and severity, imaging findings, and intensity grade of ESWT influence. The purpose of our study is to identify the predictive factors for a minimum clinically successful therapy (MCST) after ESWT for chronic plantar fasciitis.

## Materials and Methods

### Patient Selection

The study was conducted in accordance with the principles of the Declaration of Helsinki and approved by the ethics committee of Shanghai LongHua Hospital. All the participants signed informed consents prior to the study. Data of patients treated in our institution for symptomatic chronic plantar fasciitis between 2014 and 2017 were prospectively collected and analyzed.

Chronic plantar fasciitis was diagnosed based on clinical symptoms, physical examinations and imaging studies. All the patients had the typical symptoms, such as morning heel pain.

The inclusion criteria were: (1) age of >18 years; (2) having morning heel pain that relieved after a short-distance walk; (3) having local pain in the area where the fascia attaches to the heel; (4) having a symptomatic duration of >1 month. The exclusion criteria were: (1) having feet or ankles with physical dysfunction (for example, instability); (2) having infections or tumor at the heel; (3) having diabetes mellitus, peripheral vascular abnormalities; (4) having a history of trauma or calcaneal fracture.

All eligible patients completed a demographic and clinical questionnaire that assessed age, gender, BMI, affected side(bilateral, unilateral), duration of symptoms, Roles and Maudsley score (RM score) (Table 1), VAS when taking first steps in the morning, oedema, bone spurs and intensity grade of ESWT. Participants were randomly assigned by the researcher into low, moderate or high-intensity groups with a number allocation of 1:1:1 using a computer generated randomization schedule. Patients were kept blinded for the allocated treatment during the follow-up period. The statistician was also blinded.

**Table 1:** Roles and Maudsley score.

Score	Item	Content
1	excellent	no symptoms, unlimited walking ability without pain, patient satisfied with the treatment outcome
2	good	ability to walk for >1 hour without pain, symptoms decreased after treatment, patient satisfied with the treatment outcome
3	acceptable	inability to walk >1 hour without pain, symptoms somewhat improved and pain more tolerable than before treatment, patient slightly satisfied with the treatment outcome
4	poor	inability to walk without severe pain, symptoms not improved or even worsened after treatment, patient not satisfied with the treatment outcome

### Procedures

The study protocol used was in line with the current state-of-the-art in treating plantar fasciopathy with ESWT performed by the principal authors with the EMS Swiss Dolorclast^15^. All the subjects in the ESWT group received three sessions of ESWT with an interval of one week. All treatments were performed with no local anesthesia. Before the treatment, the point of maximal tenderness was clinically located by the clinician, and the ultrasound coupling gel was applied to smooth the wave transmission between the ESWT head and the skin. Shock waves (2000 times, 2.5-bar pressure, 10-Hz frequency) were transmitted to the areas of the painful heel, calcaneal insertion of the plantar fascia, and myofascial junction at the heel dorsum. In the low, moderate and high-intensity group, ESWT was applied in each treatment session (8 impulses per second, an energy flux density of 0.2 mJ/mm^2^, 0.4 mJ/mm^2^, 0.6 mJ/mm^2^, respectively). All the patients were kept blinded for the allocated treatment during the follow-up period. The statistician was blinded as well.

### Outcomes

After the ESWT, follow-up visits were scheduled. Patients’ VAS pain score changes (percentage, the lowest, the highest) after ESWT were assessed. VAS has a horizontal, 100-mm-long line, with “no pain” recorded on the left side (score: 0) and “pain as bad as it could be” on the right side (score: 10). Patients were asked to place a hatch mark on the line that corresponded to their current level of pain, both at rest and on most painful movement. The VAS score was then determined by measuring the millimeters between the left endpoint and the point that the patient marked. All patients finished a 24-week follow-up. To define the patients benefiting from ESWT, therapeutic success was considered when the VAS score decreased by ≥60% according to the baseline. The clinical outcomes were assessed by observers blind to treatment allocation^20,21^.

### Artificial neural networks

The ANN (Artificial neural networks) model was created using the SPSS 20.0 statistical software (SPSS Inc., Chicago, Illinois)^22^. ANN analysis was used to choose the influencing variables and model their effect on MCST during a 6-months follow-up. The multilayer perceptron (MLP) model was selected. The MLP model consists of three layers: input layer, hidden layer and output layer. The MLP ANN used predictive factors consisting of input layer (age, sex, BMI, affected bilateral side, duration of symptoms, RM score, VAS, intensity grade, edema, presence of heel spur) and output layer (achieving MCST or not) to define (learn) the complex relationship between inputs and outputs. The output layer consists of one neuron, the target error is 0.01, the learning rate is 0.1, the maximum training period is 2000, and the model training will be finished when the error reaches the minimum value.

Patients in our study cohort were also completely randomly allocated, 75% selected as the total training sample and 25% as the keeping sample. Then 75% of the total sample were redistributed as the training sample and 25% as the testing sample. They were used for the establishment of ANN models. Once the MLPANN was trained, it was then used to estimate the results from new input data^23,24^.

### Statistical analysis

Student *t* tests were performed for continuous variables. Categorical variables were evaluated with Fisher’s exact test or Chi-square test. *P* < 0.05 was considered statistically different. The receiver-operator characteristic (ROC) curve analysis was created and used to calculate specificities, positive predictive value and negative predictive at 95% sensitivity. When the ANN model for the achievement of MCST was built, the accuracy rate was validated by t tests for the validation group. Hosmer-Lemeshow test was used to test the goodness of fit.

## Results

### Patient demographic and clinical characteristics

A total of 210 eligible patients with chronic plantar fasciitis at our institute between 2014 and 2017 were included in the study (112 males and 98 females, with a mean age of 54.1 ± 13.6 year. All the patients provided written informed consent before enrollment and were randomized into three groups. The baseline scores at first visit, the mean of baseline RM score was 2.6 ± 0.5 points and the mean of baseline VAS sores was 6.2 ± 1.9 points. Of these patients, 76 (36.5%) presented with a BMI of <26, 112 (53.3%) of 26–30 and 22 (10.5%) of >30. The mean duration of symptoms was 84.1 days (range, 30–270 days), 56(26.7%) showing oedema and 131(62.4%) showing heel spur. There were 140 (66.7%) eligible patients who achieved the MCST. Table 2 summarizes the demographic and clinical characteristics. Table 3 shows the sample distribution of ANN model.

**Table 2:** Demographic and Clinical characteristics.

		Total 210
Age (mean ± SD), years		54.1 ± 13.6
Gender: male		112(57.1%)
BMI	<26	76(36.2%)
	26–30	112(53.3%)
	≥30	22(10.5%)
Affected bilateral side		56(26.7%)
Duration of symptoms (mean, range), days		84.1(30,270)
Roles and Maudsley score		2.6 ± 0.5
VAS		6.2 ± 1.9
Intensity Grade	low	70(33.3%)
	moderate	70(33.3%)
	high	70(33.3%)
Oedema		46(21.9%)
Presence of heel spur in X-ray		131(62.4%)

SD: standard deviation.

**Table 3:** The sample distribution of ANN model.

	Training sample	Testing sample	Keeping sample	Total
Number	118	40	52	210

### ANN model analysis

**Figure 1:**
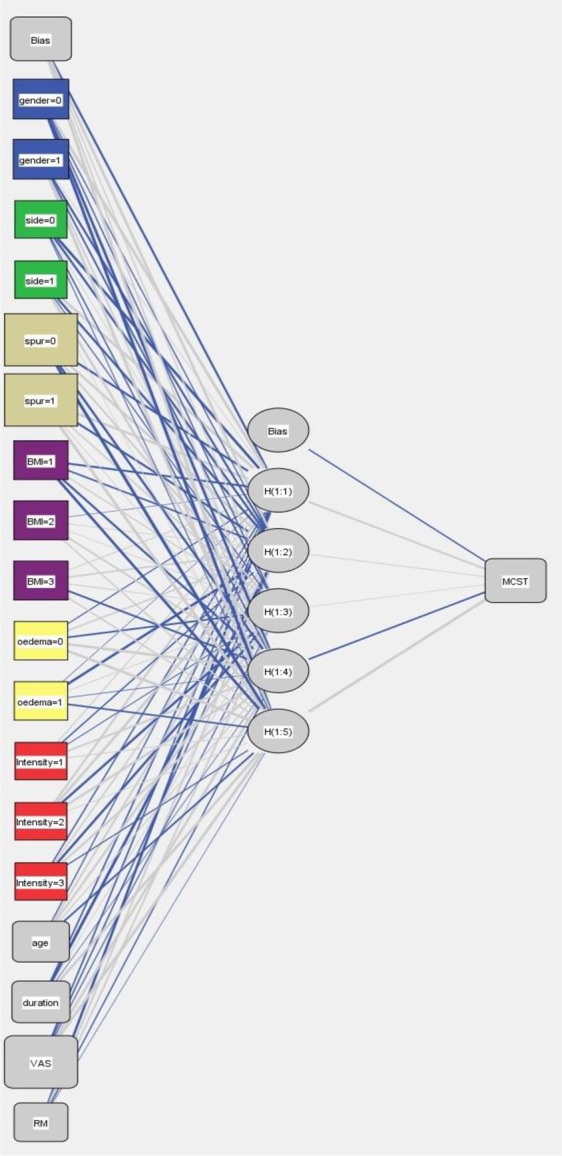
ANN model output diagram with insets for each layer.

Table 4 summarizes the 10 predictive factors. Univariate analysis of all independent factors for ESWT was done in groups with and without MSCT. Among all the 10 factors, higher VAS score (short-term increase of pain severity when the patient took first steps in the morning or had plantar fascia spur) increased the odds of MCST (p < 0.05). These three factors (VAS, presence of heel spur and duration of symptoms) were then put into the ANNs model. Importance, normalized Importance and value of the 10 independent variables are illustrated in Table 5. The interrelationships between predictor variables (input nodes), hidden variables (5 items in 1 hidden layer), and MSCT are shown in Fig. 1.

**Table 4:** Definition of the 10 predictive factors.

Variable	Definition of Neurons	Type	Value
Age	The years of old	continuous	18–79
Gender	Male/Female	categorical	0,1
Affected bilateral side	Yes/No	categorical	0,1
Duration of symptoms	The days of duration	continuous	30–270
RM score	The number of RM score	categorical	1,2,3,4
VAS	The number of VAS	continuous	0–10
BMI	Height/Weight^2^	categorical	1,2,3
Intensity grade	Low/Moderate/High	categorical	1,2,3
Oedema	Yes/No	categorical	0,1
Presence of heel spur	Yes/No	categorical	0,1

**Table 5:** Importance and value of the 10 independent variables.

No	Variable	Importance	Normalized Importance	p-value
1	VAS	0.261	100.0%	0.012*
2	Presence of heel spur	0.256	98.0%	0.022*
3	Duration of symptoms	0.098	37.6%	0.021*
4	Age	0.086	32.7%	0.231
5	Gender	0.070	26.8%	0.334
6	BMI	0.057	21.7%	0.221
7	RM score	0.053	20.3%	0.323
8	Oedema	0.050	19.2%	0.341
9	Affected bilateral side	0.035	13.3%	0.568
10	Intensity grade	0.034	12.8%	0.654

*The three most predictive value factors.

### Accuracy of the model

**Figure 2:**
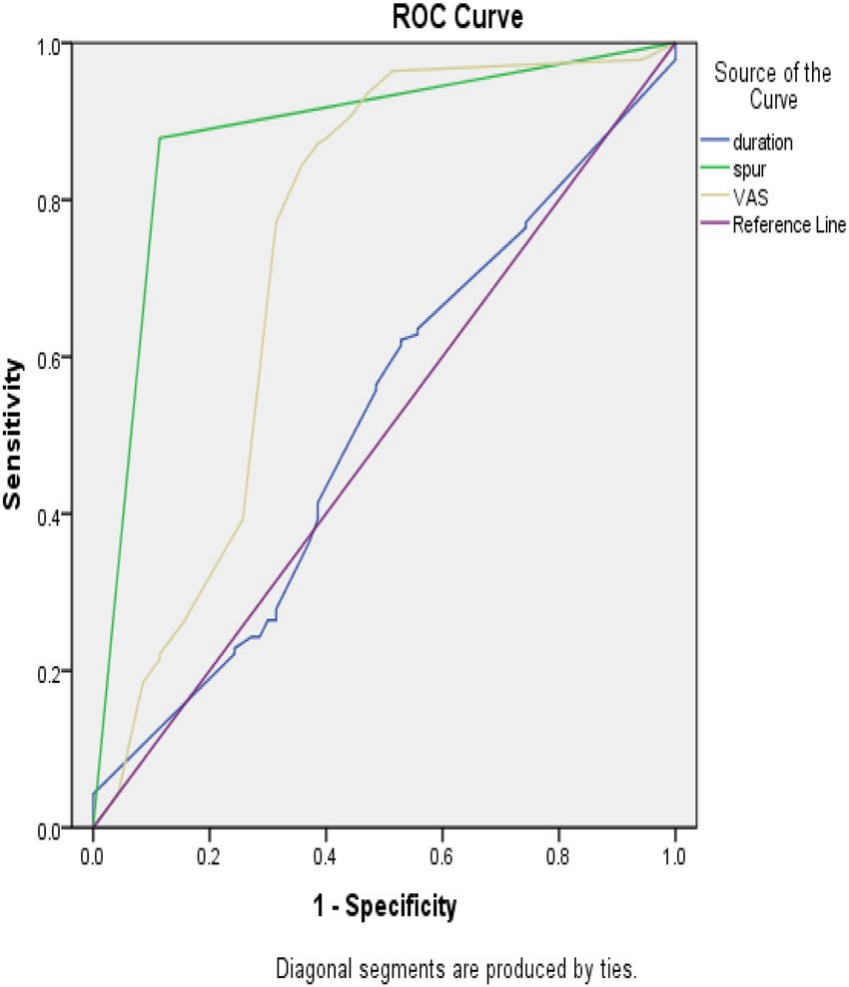
ROC curve based on ANN model contains Duration of symptom, Presence of heel spur and VAS score.

For VAS, heel spur and symptom duration, the diagnosis sensitivity was 84.3%, 87.9% and 61.4%, respectively; the diagnosis specificity was 35.7%, 37.4% and 22.3%, respectively. The result of sensitivity and specificity both demonstrated a high accuracy. The positive predictive value was 69%, 72% and 57%, respectively. The negative predictive value was 82%, 84% and 59%, respectively. The AUC was 0.738, 0.882 and 0.520, respectively, which demonstrated a good discrimination. Table 6 and Fig. 2 demonstrated the Area under ROC curve, predictive values of ANN models and three individual parameters to predict the achievement of MCST.

**Table 6:** The area under ROC curve and predictive values of ANN models and 3 individual parameters to predict the achievement of MCST.

Characteristics/Model	Sensitivity (%)	Specificity (%)	AUC (95%CI)	PPV (%)	NPV (%)
VAS	84.3	35.7	0.738(0.656–0.820)	69	82
Presence of heel spur	87.9	37.4	0.882(0.829–0.935)	72	84
Duration of symptoms	61.4	22.3	0.520(0.437–0.604)	57	59

Note: PPV, positive predictive value; NPV, negative predictive value.

The Hosmer–Lemeshow statistic assessed the goodness fit of ANN model, showing a good fitting with an overall accuracy of 92.5% (Table 7).

**Table 7:** Predictive model established by ANN model (n = 210).

Observed	Predicted		
	Not Achieved MSCT	Achieved MSCT	Percentage Correct
Not Achieved MSCT	63	7	90.0%
Achieved MSCT	7	133	95.0%
Overall Percentage			92.5%

Note: χ^2^ = 6.635, df = 8, P = 0.577, overall accuracy 92.5%.

## Discussion

Data mining (DM) is a mathematical process to extract the potentially useful information from a fuzzy and incomplete data. DM not only analyzes, synthesizes, and deducts the data, but also helps decision makers in actual situation with its exploratory and predictive function. Integrating artificial intelligence and computer technology, statistics and medical science, medical DM is widely used in disease diagnosis, disease related factors analysis, disease prediction, medical imaging and so on^22,25–27^.

In medicine, logistic regression (LR) model is a widely used DM technique to predict the curative effect of treatment^28^. A disease usually has a complicated pathogenesis that is regulated by diverse factors. The LR model established with traditional statistical methods has a low efficiency to process data, especially those non-independent influence factors. But, ANN model has a strong power to process nonlinear data. ANN model consists of two processes: forward propagation and error reverse propagation. The two processes are carried out continuously, which is the ANN process of the learning and training to reach the acceptable level.

ANN is not new but still underutilized in rehabilitation medicine. Despite being helpful in double-checking and routine examinations, ANN cannot replace medical experts in decision-making. In this study, the results analyzed by ANN model were more precise and rigorous than those by our previous study^29^. ANN model can be considered accurate and reliable in predicting MCST for its overall accuracy of 92.5%, which is much higher than that of multiple stepwise logistic regression model.

The diagnosis of plantar fasciitis is based on the patient’s history and clinical examination. The patients often reports a gradual onset of inferior heel pain that worsens with the first steps in the morning or after a long-term standing. After movements are made, the pain tends to subside but worsens again by the end of the day. Various management strategies have been developed to treat plantar fasciitis. Nonsurgical treatment is the mainstay. Extracorporeal shock wave therapy is an increasingly welcomed therapeutic approach to treat chronic and recalcitrant plantar fasciitis, with a success rate up to 34–88%. ESWT is a lithotripsy technology that target shock waves to the plantar fascia area. The shock wave can be generated through three approaches: electrohydraulic, electromagnetic, and piezoelectric^30^.

We investigated the prognostic factors influencing MCST three months after ESWT for patients with chronic plantar fasciitis. ANN model was useful to guide physiotherapist in recommending ESWT with a high predictive ability and anticipating the outcome based on patients’ characteristics. The results revealed that the VAS score before ESWT was the most influential factor deciding the odds of successful MCST.

The mechanism of analgesia in ESWT is still not clear. Some theories have been put forward to explain it. Some scholars believe that shock wave energy that concentrated on unit area is described by energy flux density, shows the shock wave stream which is reflected perpendicularly to the source of production. Pressure waves of ESWT pass through fluids and soft tissues, such as superficial fascia, tendons, ligaments, and pose their effects on areas of impedance changes. Sometimes at cellular level, they mechanically promote microcirculation and relieve the pain. Of these effects, the strongest one is the transient damage to neuronal cell membrane or the increase of pain nociceptors^31^. Another scholars think that ESWT can press powerful shock waves on local soft tissues to intensely stimulate nerve ends and inhibit the action of the pain factors. When the pain receptors are continuously and intensely stimulated, the sensitivity and compliance of pain nerve ends reduce.

The formation of heel spur is a continuous biological process. The plantar fascia is a critical structure arising from the medial process of the calcaneal tuberosity and inserting into the digits of the foot. Heel spur consists of mature lamellar bone and demonstrates degeneration and fibro-cartilaginous proliferation, even ossification in the condition of plantar fasciitis^32–34^. At present, the relationship between the plantar calcaneal spurs and the plantar fasciitis pain stills remains elusive. McMillan compared the clinical characteristics of asymptomatic and symptomatic subcalcaneal spur groups, finding a strong association between chronic heel pain and heel bone spurs^35^. Researchers have also found that the degree of pain and the length of spurs are positively correlated (the longer the length of spurs, the severer the pain)^36,37^. Furthermore, the direction and length of subcalcaneal spurs can affect the occurrence and prognosis of plantar fasciitis^36^. So we hold the view that attention should be paid to calcaneal spurs in the diagnosis and treatment of plantar fasciitis. In our study, we found that the prevalence of calcaneal spurs in X-ray significantly affected the efficacy of ESWT. Some other studies also showed that ESWT had a significant effect on symptomatic heel spurs without any irreversible side effect^38^. Cosentino found that ESWT could relieve plantar heel pain symptoms and modify the structre of heel spurs ultrasonographically and radiographically^39^. The mechanism may be that ESWT can break sclerotic bones by producing microfissures and bony fragments^40^. But we didn’t draw a subgroup analysis to analyze the relationship between the clinical efficacy of ESWT and the classification of calcaneal spurs.

In this study, we also revealed that high BMI was a prognostic factor positively influencing the clinical efficacy of ESWT. Notarnicola analyzed 355 patients receiving ESWT for rotator cuff tendinitis, epicondylitis, Achilles tendinopathy, trocanteritis, jumper’s knee or plantar fasciitis, discovering that male gender and high BMI are prognostic factors of a successful ESWT. High BMI is also a risk factor in the onset of plantar fasciitis, which is contradictory and misleading. Some theories can explain. The diffusion of shockwaves follows the physical laws in the reflection and absorption of the acoustic waves, which are affected by the characteristics of the medium and the diversity in density, and the impedance of the skin, fat, muscle and bone. Adipose tissues, presenting an acoustic impedance close to that of water, allow acoustic waves to pass and reach their underlying tissues^41,42^. Some biological theories can also be put forward to explain the result of this study. Adipose tissues are rich in stem cells. ADSCs (adipose-derived stem cells) could be isolated using automatic centrifuge for cell isolation specialized in cells from adipose tissue enhancing the bio-stimulating effects of the ESWT therapy^43^.

This study is innovative in the following aspects. First, to our knowledge, we were the first group to demonstrates that an ANN is a sensitive and specific regression model in predicting the 6-months’ follow-up efficacy of ESWT for chronic plantar fasciitis. Our previous publications show that ESWT is an effective and safe tool in the treatment of chronic plantar fasciitis and should be an alternative to surgery^44^. Second, it finds that high VAS score (symptomatized by severer pain on taking first steps in the morning, edema, and heel spur) significantly affected the outcome of the treatment^29^. In this study, we conducted a more reasonable and accurate statistical analysis to guarantee the comprehensiveness by ANN model. And the follow-up is longer than before.

There are some limitations in our study. First, the small sample size (210) is adequate to evaluate the variables for ANN model (at least 5 times than the number of factors), but as a data mining study, the sample size should be broadened to explore and verify its effectiveness. Second, we did not identify all possibly significant variables to predict the efficacy of ESWT. Future studies using ANN model should contain more input factors (such as the interval, impulses per second and so on) and other detailed result data. Third, the time of follow-up was relatively short. Lacking long-term follow-ups, its predictive factors for long-term efficacy remain unknown. Last, the study didn’t compared the ANNs model with other regression models to assess the accuracy.

## Conclusions

Higher VAS symptomatized by short-duration severer pain on taking first steps in the morning or plantar fascia spur are important prognostic factors for the efficacy of ESWT. The ANN predictive model is reasonable and accurate in investigating their influence on MCST. These parameters analyzed by a predictive model can help the decision-making for the application of ESWT.

## Data Availability

All supporting data can be provided upon request to the authors.
